# Soy Isoflavones Accelerate Glial Cell Migration *via* GPER-Mediated Signal Transduction Pathway

**DOI:** 10.3389/fendo.2020.554941

**Published:** 2020-11-04

**Authors:** Winda Ariyani, Wataru Miyazaki, Izuki Amano, Kenji Hanamura, Tomoaki Shirao, Noriyuki Koibuchi

**Affiliations:** ^1^ Research Fellow of Japan Society for the Promotion of Science, Tokyo, Japan; ^2^ Department of Integrative Physiology, Graduate School of Medicine, Gunma University, Maebashi, Japan; ^3^ Department of Bioscience and Laboratory Medicine, Graduate School of Health Science, Hirosaki University, Hirosaki, Japan; ^4^ Department of Neurobiology and Behavior, Graduate School of Medicine, Gunma University, Maebashi, Japan

**Keywords:** genistein, daidzein, S-equol, 17β-estradiol, astrocyte, F-actin, development, migration

## Abstract

Soybean isoflavones, such as genistein, daidzein, and its metabolite, S-equol, are widely known as phytoestrogens. Their biological actions are thought to be exerted *via* the estrogen signal transduction pathway. Estrogens, such as 17β-estradiol (E2), play a crucial role in the development and functional maintenance of the central nervous system. E2 bind to the nuclear estrogen receptor (ER) and regulates morphogenesis, migration, functional maturation, and intracellular metabolism of neurons and glial cells. In addition to binding to nuclear ER, E2 also binds to the G-protein-coupled estrogen receptor (GPER) and activates the nongenomic estrogen signaling pathway. Soybean isoflavones also bind to the ER and GPER. However, the effect of soybean isoflavone on brain development, particularly glial cell function, remains unclear. We examined the effects of soybean isoflavones using an astrocyte-enriched culture and astrocyte-derived C6 clonal cells. Isoflavones increased glial cell migration. This augmentation was suppressed by co-exposure with G15, a selective GPER antagonist, or knockdown of GPER expression using RNA interference. Isoflavones also activated actin cytoskeleton arrangement *via* increased actin polymerization and cortical actin, resulting in an increased number and length of filopodia. Isoflavones exposure increased the phosphorylation levels of FAK (Tyr397 and Tyr576/577), ERK1/2 (Thr202/Tyr204), Akt (Ser473), and Rac1/cdc42 (Ser71), and the expression levels of cortactin, paxillin and ERα. These effects were suppressed by knockdown of the GPER. Co-exposure of isoflavones to the selective RhoA inhibitor, rhosin, selective Cdc42 inhibitor, casin, or Rac1/Cdc42 inhibitor, ML-141, decreased the effects of isoflavones on cell migration. These findings indicate that soybean isoflavones exert their action *via* the GPER to activate the PI3K/FAK/Akt/RhoA/Rac1/Cdc42 signaling pathway, resulting in increased glial cell migration. Furthermore, *in silico* molecular docking studies to examine the binding mode of isoflavones to the GPER revealed the possibility that isoflavones bind directly to the GPER at the same position as E2, further confirming that the effects of the isoflavones are at least in part exerted *via* the GPER signal transduction pathway. The findings of the present study indicate that isoflavones may be an effective supplement to promote astrocyte migration in developing and/or injured adult brains.

## Introduction

Soybean isoflavones are a natural class of isoflavones, exclusively produced by the legume family (1). They are well-known phytoestrogens that can bind and modulate the action of nuclear receptors including estrogen receptor (ER), thyroid hormone receptor, androgen receptor, pregnane X receptor, and aryl hydrocarbon receptor (2–6). Binding of isoflavones to receptors exerts various effects at the molecular, cellular, and organ levels (7). In addition, isoflavones also can affect other pathways by modulating membrane receptors, protein kinases, transcription factors, chromatin remodeling, antioxidants, and altering some enzyme activities (8, 9). Genistein, daidzein, and S-equol, a metabolite of isoflavones, are the main isoflavones that have been intensively studied. This wide variety of actions indicates that isoflavones act *via* several different signaling pathways.

Recent studies have shown that 17β-estradiol (E2) activates the G protein-coupled estrogen receptor (GPER; also known as GPR30), which then initiates several intracellular signal transduction pathways, such as the epidermal growth factor receptor-mediated pathway to activate extracellular signal-regulated kinase 1/2 (ERK1/2) and/or Akt-mediated pathways (10–13). In addition to E2, isoflavones may also interact with the GPER. *In vitro*, activation of the GPER by isoflavones has been demonstrated to trigger cell signaling pathways and growth factor receptor cross-talk (14, 15). Our previous study showed that S-equol could activate GPER to increase p-ERK1/2 leads to induced proliferation, growth, and differentiation in both neurons and astrocytes during cerebellar development (14). The K_d_ (dissociation constant) of E2 to the GPER is 3–6 nM. Meanwhile the effective concentration 50 (EC_50_) values of isoflavones to the GPER based on functional dose-response 133 nM for genistein, < 1 nM for daidzein, and 100 nM for S-equol (16). Based on these findings, we hypothesized that isoflavones would affect the GPER signaling pathway and alter cellular function.

Estrogen plays a key role in the development and functional maintenance of the central nervous system (CNS) through genomic (*via* the ER) and rapid nongenomic responses *via* the GPER (17, 18). GPER is highly expressed in the CNS, including glial cells (19). GPER knockout mice showed altered anxiety levels and stress response (18), and this phenotype could not be fully rescued by estrogen treatment (18). These results indicate the involvement of the GPER in the normal development of the CNS. However, the role of the GPER on the function of each subset of cells remains unclear.

Glial cells are essential for brain functioning during development and in the adult brain and have been shown to play a significant role in neuronal migration, proliferation, differentiation, and synaptogenesis (20). Glial cells comprise astrocytes, oligodendrocytes, and microglia, among which, astrocytes are the most abundant cell type in the CNS (21). Astrocytes are most likely migrate to their final destination shortly after their birth in the ventricular zone or subventricular zone, cortical gray matter astrocytes were found to migrate along with radial glia processes, whereas white matter astrocytes migrated along developing axons of neurons (21, 22). Astrocytes are activated in injured or diseased CNS and begin to proliferate and migrate. This process is known as astrogliosis (21). High levels of GPER expression in astrocytes may affect the physiological response of astrocyte during development or in the adult brain.

Cell migration is a critical process in both physiological and pathological processes. The Rho family of GTPase is the core regulator of cell migration (23). In the CNS, Rho GTPase family members, such as RhoA, Rac1, and Cdc42, play fundamental roles in a wide variety of cellular processes, including rearrangement of the actin cytoskeleton, cell polarity, and controlling dynamic astrocyte morphology (24–26). Deletion of Rac1 and Rac3 in cerebellar granule neurons (CGNs) led to severe impairment of radial migration of CGNs, defects in the internal granule layer, and decreased cerebellum size (27). Cdc42 knockout mice also showed impaired radial migration of CGNs, disturbed alignment of Bergmann glia in the Purkinje cell layer, and aberrantly aligned Purkinje cells (24). In addition, astrocytes lacking Cdc42 were still able to form protrusions, although were unable to migrate in a directed manner toward the scratch/wound (26). Since isoflavones may bind to the GPER in astrocytes, these results raise the possibility that isoflavones affect astrocyte migration *via* the RhoGTPase signaling pathway.

Our previous study showed that S-equol, a daidzein metabolite, activates GPER to induced F-actin rearrangement lead to increase astrocyte migration during cerebellar development with unknown mechanisms (14). The present study examined the effects of isoflavones on cell migration of glial cells using astrocyte-enriched cultures of cerebral cortex and astrocyte-derived C6 clonal cells by wound healing and cell migration/invasion assays. We also examined changes in the actin cytoskeleton by labeling F-actin using phalloidin. Our findings revealed that isoflavones induced F-actin rearrangement and accelerated cell migration. These effects were reduced by the GPER inhibitor, G15, or short interfering RNA (siRNA) knockdown of GPER. Furthermore, activation of GPER by isoflavones activated the PI3K/Akt signaling pathway that induce RhoGTPase to accelerate cell migration. The results of our *in silico* molecular docking study revealed a common possible binding site of the isoflavones on the GPER.

## Material and Methods

### Chemicals

Genistein, daidzein, and E2 were purchased from Sigma (St. Louis, MO, USA). S-equol, G-15, casin, ML-141, LY294002, and U0126 were purchased from Cayman Chemical (Ann Arbor, MI, USA). Rhosin HCl was purchased from Tocris Bioscience (Avonmouth, Bristol, UK). The purity of all chemicals was >98%.

### Clonal Cell Culture

C6 rat glioma clonal cells were maintained in Dulbecco’s modified Eagle’s medium (DMEM) supplemented with 10% fetal bovine serum (FBS) and antibiotics (100 U/ml penicillin and 100 µg/ml streptomycin) at 37°C with 5% CO_2_. The serum was stripped of hormones by constantly mixing with 5% (w/v) AGX1-8 resin (Bio-Rad, Hercules, CA, USA) prior to ultrafiltration (28).

### Primary Culture of Mouse Cerebral Cortex Astrocytes

The animal experimentation protocol in the present study was approved by the Animal Care and Experimentation Committee, Gunma University (19-024, 17 December 2018), and all efforts were made to minimize animal suffering and the number of animals used.

A primary culture of mouse cerebral cortex astrocytes was prepared as previously described (29, 30) with slight modifications. A pregnant C57BL/6 strain mice were purchased from Japan SLC (Hamamatsu, Japan). Briefly, postnatal day 1 mouse cerebral cortices were dissected and digested with 2.5% trypsin (Wako, Japan) in Hank’s balanced salt solution (Wako) for 30 min with continued shaking at 37°C. Cells were resuspended in an astrocyte culture medium (high-glucose DMEM, 10% heat-inactivated FBS, and 1% penicillin/streptomycin), and 10–15 million cells were plated on 10-cm dishes coated with Collagen I (Iwaki, Japan). Cells were incubated at 37°C in a CO_2_ incubator. On day 3 *in vitro* (DIV3), astrocyte culture medium was replaced with phosphate-buffered saline (PBS). Dishes were then shaken by hand for 30–60 s until only the adherent monolayer of astrocytes was left. The PBS was then replaced with a fresh astrocyte culture medium. Astrocytes were harvested on DIV7 using 0.25% trypsin 1 mM disodium EDTA (Wako), and then plated on 12 or 24 well dishes. Cells were used for cell invasion assay or F-actin staining.

### In Vitro Wound Healing (Scratch) Assay

C6 cells were plated in 24-well plate and cultured until confluent. Prior to making a scratch, cells were serum-starved in FBS-free DMEM for 6 h. A wound was created by scratching the monolayer with a 200-µl pipette tip. Floating cells were washed away using PBS. Serum-free DMEM and/or isoflavones, E2, G15, U1026, LY294002, rhosin, Casin, and/or ML-141 were added to the wells and incubated for a further 24 h. At 0 and 24 h, live-cell staining was performed using Cellstain-Hoechst 33258 solution (Dojindo Molecular Technologies, Inc., Japan) according to the manufacturer’s protocol. Images of the scratched area were taken at 0 and 24 h. The cells were then visualized using a fluorescence microscope (Keyence BZ9000, Keyence Corporation of America, Itasca, IL, USA). Cell migration was determined at the edges of the wound, and the percentage migration was determined as the ratio between migrated distance and initial distance of the wound.

### Matrigel Invasion Assay

*In vitro* invasion assays were performed using a 24-well Millicel hanging cell culture insert and a Corning Matrigel matrix according to the manufacturer’s instructions. In brief, astrocytes were seeded at a density of 1 × 10^5^/ml in serum- free DMEM in the upper chamber. The lower chamber was filled with serum-free DMEM and/or isoflavones, E2, G15, U1026, LY294002, rhosin, casin, and/or ML-141. After 16–18 h of incubation, noninvading cells in the upper chamber were removed with a sterile cotton swab. The filters from the inserts were fixed with 4% paraformaldehyde (PFA) and stained with DAPI. The cells were then inspected using a laser confocal scanning microscope (Zeiss LSM 880, Carl Zeiss Microscopy GmbH, Jena, Germany). The number of invaded cells on the lower surface of the filter was counted.

### Filopodia Formation and Cortical F-Actin Score Index

Astrocytes were cultured on poly-L-lysine-coated coverslips and serum-starved DMEM for 24 h. The cells were then treated with either isoflavones or E2 for 30 min then washed with PBS and fixed with 4% PFA followed by blocking with 2% FBS. The cells were incubated with CytoPainter Phalloidin-iFluor 594 reagent (Abcam, Cambridge, UK) and nuclei were stained with DAPI and then visualized under a laser confocal scanning microscope (Zeiss LSM 880, Carl Zeiss Microscopy GmbH). The degree of cytoskeletal rearrangement was examined using the FiloQuant by ImageJ Fiji (NIH) or cortical F-actin score CFS index (31). The CFS index was determined based on at least three independent experiments. Briefly, F-actin cytoskeletal reorganization for each cell was scored on a scale ranging from 0 to 3, based on the degree of cortical F- actin ring formations 0, no cortical F-actin, normal stress fibers; 1, cortical F-actin deposits below half the cell border; 2, cortical F-actin deposits exceeding half the cell border; and 3, complete cortical ring formatting and/or total absence of central stress fiber. A minimum of 50 cells were examined from each group in each independent experiment, and the CFS index for treated astrocytes was the average score of the counted cells ± standard error of the mean (SEM).

### Immunocytochemistry Analysis of Protein Phosphorylation and F-Actin Formation

Cultured cells were exposed to isoflavones or E2 for 30 min then rinsed three times with PBS, fixed with 4% PFA, and blocked with 2% FBS. Cells were then incubated with rabbit monoclonal anti-phospho-Akt (Ser473) (D9E) XP (1:200; Cell Signaling, MA, USA), anti-phospho-p44/42 MAPK (ERK1/2) (Thr202/Tyr204) (1:200; Cell Signaling), or anti-phospho-Rac1/Cdc42 (Ser71) (1:200; Cell Signaling) antibodies, followed by CytoPainter Phalloidin-iFluor 594 reagent (Abcam) and donkey anti-rabbit IgG (H+L) secondary antibodies, Alexa Fluor^®^ 488 conjugate (1:200; Thermo Fisher Scientific, Inc, Waltham, MA, USA). Cell nuclei were also stained with DAPI. The cells were then inspected using a laser confocal scanning microscope (Zeiss LSM 880, Carl Zeiss Microscopy GmbH).

### RNA Interference Assay

Astrocyte-enriched cultures were transfected with siRNAs for ERα (Thermo Fisher Scientific.), ERβ (Thermo Fisher Scientific), GPER (Integrated DNA Technologies, Inc., Coralville, IA, USA), or negative control RNAs (nontargeting control [catalog no. SIC001; Sigma-Aldrich] or negative control DsiRNA [catalog no. 51-01-14-03; Integrated DNA Technologies, Inc.]), using lipofectamine RNAiMAX reagent (Thermo Fisher Scientific) according to the manufacturer’s protocol. The list of siRNA sequences used in this study is listed in **Table 1**. Briefly, siRNA lipid complexes [1 nM of control siRNA (scrambled RNA), ERα, ERβ or GPER siRNA] were incubated for 20 min, and then added to astrocytes at approximately 80% confluency in 35-mm dishes. After 16–24 h, the cells were subjected to matrigel invasion assay. The efficacy of the siRNA knockdown was verified by quantitative real-time PCR (qRT-PCR). Total RNA was extracted using *SuperPrep* cell lysis and RT kit for qPCR reagent (TOYOBO Bio-Technology, Japan) according to the manufacturer’s instructions. qRT-PCR was performed using THUNDERBIRD SYBR qPCR mix (TOYOBO) as per the manufacturer’s instructions and using a StepOne RT-PCR System (Thermo Fisher Scientific). The list of primers used in this study is listed in the supplementary information 1 (SI. 1). qRT-PCR was performed as follow: denaturation at 95°C for 20 s, followed by amplification at 95°C for 3 s and at 60°C for 30 s (40 cycles). All experiments were repeated three times, using independent RNA preparations to confirm the consistency of the results. All mRNA levels were normalized to that of Gapdh.

**Table 1:** List of short interfering RNA (siRNA) sequences.

	Sequences	
**ERα**	Sense (5’-3’)	CGUCAAGUCGGUUCCGCAUGAUGAA
	Antisense (5’-3’)	UUCAUCAUGCGGAACCGACUUGACG
**ERβ**	Sense (5’-3’)	GCGUGGAAGGGAUUCUGGAAAUCUU
	Antisense (5’-3’)	AAGAUUUCCAGAAUCCCUUCCACGC
**GPER**	Sense (5’-3’)	GUGUUCAACCUGGACGA
	Antisense (5’-3’)	AGUACUGCUCGUCCAGGU

### Western Blot Analysis

Cultured cells were homogenized in RIPA buffer (Cell Signaling) and protease inhibitors (Complete; Roche, IN, USA). Protein concentration was measured using the Bradford protein assay (Bio-Rad) according to manufacturer’s instruction. After boiling for 5 min, protein samples (5 µg) were subjected to 5%–20% SDS-polyacrylamide Supersep Ace (Wako) gel electrophoresis, and the separated products were transferred to nitrocellulose membranes. Membranes were blocked with 5% nonfat dry milk in Tris-buffered saline containing 0.1% Tween 20, followed by an overnight incubation with the appropriate diluted primary antibodies for pFAK (1:1,000; Cell Signaling), FAK(1:1,000; Cell Signaling), pERK1/2 (1:1,000; Cell Signaling), ERK1/2 (1:1,000; Cell Signaling), pAkt (1:1000; Cell Signaling), Akt (1:1,000; Cell Signaling), pRac1/Cdc42 (1:1,000; Cell Signaling), Rac1/Cdc42 (1:1,000; Cell Signaling), Talin-1 (1:1,000; Cell Signaling), Vinculin (1:1,000; Cell Signaling), α-Actinin (1:1,000; Cell Signaling), Paxillin (1:1,000; Cell Signaling), ER-α (1:1,000; Abcam), Cortactin (1:1,000; Merck Milipore), GAPDH (1:1,000; Proteintech, IL, USA), and β-actin (1:5,000; Cell Signaling). After washing with Tris-buffered saline containing 0.1% Tween 20, membranes were incubated with horseradish peroxidase-conjugated anti-rabbit or anti-mouse IgG secondary antibody (1:3,000; Cell Signaling) for 1 h at room temperature and detected using an ECL detection system (Wako). GAPDH or β-actin were used as loading controls.

### In Silico Analysis of Ligand-Receptor Binding

*In silico* molecular docking analysis was performed as described previously (3), with slight modifications. All *in silico* calculations were performed using Dell XPS 8930 with Intel Core i7–8700 CPU @ 3.2 GHz, 16 GB DDR4 2666 MHz, NVIDIA GeForce GTX 1060 6 GB, running on a windows 10 professional operating system. Molecular structures for genistein (PubChem CID 5280961), daidzein (PubChem CID 5281708), S-equol (PubChem CID 91469), and E2 (PubChem CID 5757) were downloaded from PubChem (https://pubchem.ncbi.nlm.nih.gov/) in sdf format. The encoding sequence for GPER was retrieved from UniProt database (accession no Q6FHU6) and FASTA format was submitted to I-TASSER website (https://zhanglab.ccmb.med.umich.edu/I-TASSER/), a specialized server for building three-dimensional (3D) models of seven-transmembrane domain receptors (32–34). The I-TASSER server yielded five models from 10 different templates (3oduA, 4mbsA, 4n6hA2, 4yayA, 5nddA, 5t1aA, 5vblB, 5zbhA, 6d26A, and 6me6A). The protein conformation was refined through molecular dynamics (MD) simulations performed with GROMACS package (35). The three-dimensional structure of GPER and ligands files were opened and modified with Discovery Studio structure-based design software, version 4.0 (BIOVIA/Accelrys Inc., San Diego, CA, USA). Water molecules and other substructures (bound molecules/ligand molecules) were removed from the coordinate file before docking. GPER models 1–5 were used for the docking of genistein, daidzein, S-equol or E2. Polar hydrogen atoms were added to the 3D structure of the GPER and generated input file in pdbqt format of GPER using AutoDockTools of MGLTools (http://autodock.scripps.edu/resources/adt). Docking coordinates were determined through a grid box in PyRx–Python Prescription 0.8 Virtual Screening software for Computer-Aided Drug Design (http://pyrx.sourceforge.net/) with AutoDock 4 and AutoDock Vina are used as a docking software (36). A blind docking strategy was utilized to include all possible ligand binding sites. MD simulations of the molecular complexes were carried out for each starting pose by using the AMBER ff99SB-ILDN force field (37) for the protein and GAFF (38) for the ligand. After an initial period of equilibration, conformational sampling was performed in the isobaric-isothermal ensemble in explicit water for 10 ns, with Cl-counterions added to obtain an overall neutral system. The system was first equilibrated for 2.5 ns and structures were afterwards sampled every 0.5 ns to evaluate the binding energy and the ligand location. At the end of the MD simulations, the binding modes and the affinity of the ligands were estimated from the structures of the protein–ligand complexes obtained every nanosecond. The binding energy was evaluated by using the AutoDock Vina energy evaluation function in score-only mode. LigPlot^+^ v.1.4 (http://www.ebi.ac.uk/thornton-srv/software/LigPlus/) was used to determine the interactions existing for the GPER and ligands complexes with best affinity score values. Binding affinity was expressed as a binding free energy (kcal/mol).

### Statistical Analysis

Data are expressed as mean ± SEM of three individual experiments performed in triplicate. One-way or two-way ANOVA followed by Bonferoni’s multiple comparison tests were performed using GraphPad Prism version 8.3.1 for windows (GraphPad Software, San Diego, USA, www.graphpad.com). All *p*-values < 0.05 were considered statistically significant.

## Results

### Isoflavone Increased Cell Migration

**Figure 1:**
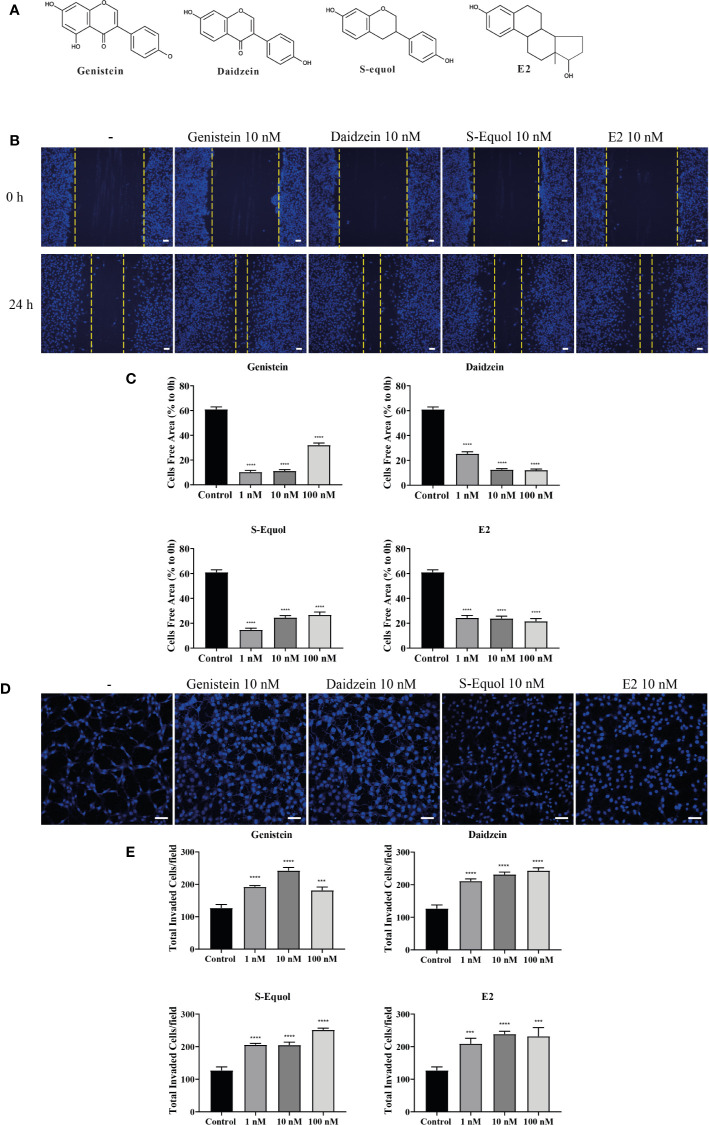
Effects of isoflavones on two-dimensional (2D) or three-dimensional (3D) cell migration. **(A)** Chemicals structures of isoflavones and E2. **(B) **Representative photomicrographs showing the effects of isoflavones on 2D wound healing assays using C6 cells. Live-cell staining was performed using Cellstain-Hoechst 33258 (Dojindo Molecular Technologies, Inc., Japan). **(C)** Quantitative analysis of the effect of isoflavones or E2 (1 – 100 nM) on cell migration measured by wound healing assay. **(D)** Representative photomicrographs showing the effects of isoflavone on 3D matrigel invasion assays using astrocytes. Cell nuclei were stained with DAPI. **(E)** Quantitative analysis of the effect of isoflavones or E2 (1 – 100 nM) on cell invasion measured by matrigel invasion assay. The total number of cells was quantified using ImageJ software (NIH). Bars represent 50 μm. Data are expressed as the mean ± SEM (n = 30 determinations) of at least three independent experiments. ^****^ *p* < 0.0001, ^***^ *p* < 0.001, indicates statistical significance measured using Bonferroni’s test compared with the control (−).

Extensive research using flat, two-dimensional (2D) glass and plastic cell migration analyses has elucidated the detailed molecular and biophysical mechanisms of the migrating process in cultured cells. However, most cells migrating through tissues undergo 3D migration under the physical constraints of the surrounding cells and extracellular matrix (39, 40). Therefore, we examined the effects of isoflavones or E2 (**Figure 1A**) in 2D and 3D migration using wound healing and invasion assays, respectively.

Representative photomicrograph images of cells stained using live-cell Hoechst staining in wound healing assays with 10 nM of isoflavones or E2 for 24 h are shown (**Figure 1B**). In the wound healing assay, genistein, daidzein, S-equol and E2 increased cell migration of C6 astrocyte clonal cells without an evident concentration dependency. Genistein accelerated cell migration at concentrations of 1 and 10 nM, whereas this effect decreased at 100 nM. Daidzein accelerated cell migration in a concentration-dependent manner and reached a peak at 100 nM. S-equol (1 nM) showed the greatest acceleration (**Figure 1C**). These results indicate that isoflavones accelerated 2D cell migration, but, was independent concentration.

We also examined 3D astrocyte migration using an invasion assay. Representative photomicrographs of invaded astrocytes stained with DAPI after isoflavones or E2 exposure are shown (**Figure 1D**). Isoflavones and E2 exposure accelerated the astrocyte migration in a dose-dependent manner, except genistein, which showed greatest acceleration at 10 nM (**Figure 1E**). These results indicate that isoflavones and E2 can induce cell migration in C6 clonal cells and astrocytes.

### Isoflavones Increased Cell Migration *via* the GPER Pathway

**Figure 2:**
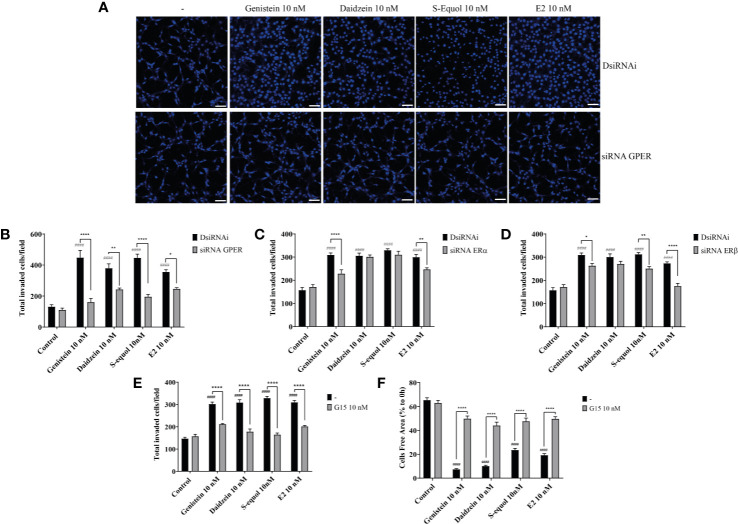
Isoflavones increased astrocyte migration *via* G-protein-coupled estrogen receptor (GPER) activation. Mouse primary cerebellar astrocytes were cultured for seven days prior to siRNA transfection, and matrigel invasion assay followed by DAPI staining was performed **(A–D)**. **(A)** Representative photomicrographs showing the effects of isoflavones on cell migration after deletion of GPER. (B–D) Quantitative analysis of the effect of isoflavones on cell invasion after deletion of GPER **(B)**, ERα **(C)**, or ERβ **(D)**. **(E)** Quantitative analysis of the effect of G15, a GPER inhibitor, on isoflavone-accelerated cell invasion using astrocytes. **(F)** Quantitative analysis of the effect of G15 on isoflavone-accelerated C6 cell migration. Bars represent 50 μm. Data are expressed as mean ± SEM (*n* = 15 determinations) and are representative of at least three independent experiments. ^####^ *p* < 0.0001, indicates statistical significance measured using Bonferroni’s test compared with the control. ^****^ *p* < 0.0001, ^**^ *p* < 0.01, ^*^ *p* < 0.05, indicates statistical significance measured using Bonferroni’s test.

Isoflavones are known phytoestrogens that activate the estrogen-mediated signaling pathway *via* nuclear ER and GPER. To examine whether further isoflavones affect cell migration, *via* ER and GPER, we used siRNA against ERα, ERβ, or GPER to knockdown their RNA expression. Knock down of GPER in astrocytes significantly reduced isoflavone and E2-accelerated cell migration (**Figures 2A, B** and **Figure SI.1**). On the other hand, knock down of ERα also significantly but weakly reduced genistein or E2 accelerated cell migration (**Figure 2C** and **Figure SI.1**). Knock down of ERβ also weakly reduced S-equol or E2-accelerated cell migration (**Figure 2D** and **Figure SI. 1**). Furthermore, co-exposure with the GPER inhibitor, G15 (10 nM), significantly reduced isoflavone or E2-accelerated cell migration in cell invasion (**Figure 2E**) and wound healing (**Figure 2F**) assays. These results indicate that isoflavones and E2 accelerate cell migration mainly *via* activation of GPER.

### Acceleration of Cell Migration by Isoflavones Was Associated With F-Actin Induction

**Figure 3:**
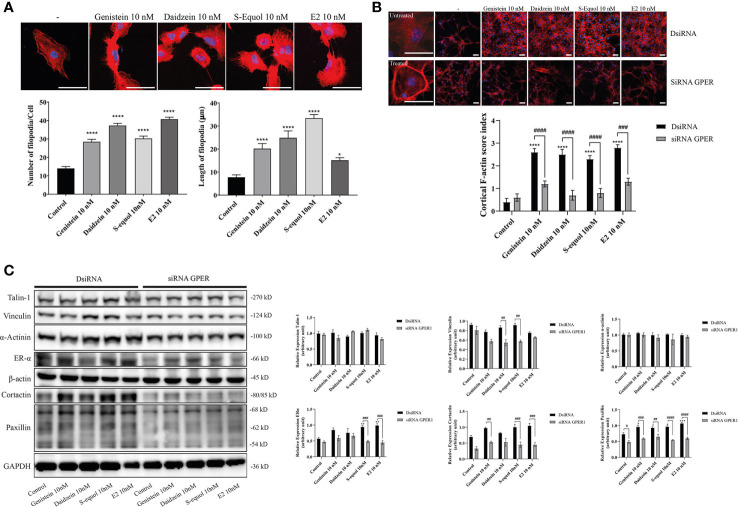
Effects of isoflavones on F-actin formation in astrocytes. Mouse primary cerebral cortex astrocytes were cultured for seven days, followed by fluorescence labeling analysis using phalloidin F-actin and DAPI. **(A)** Upper panel: Representative photomicrographs showing the F-actin and DAPI staining to examine the effects of isoflavones and E2 on filopodia formation. Lower panel: Changes in the number and length of filopodia. The number and length of filopodia were quantified using FiloQuant Fiji ImageJ software (NIH). **(B)** Upper panel: Representative photomicrographs showing F-actin and DAPI staining to examine the effects of isoflavones and E2. The first column shown the overview images with higher magnification to differentiate between the treated and untreated cells. Astrocytes were transfected with DsiRNA or siRNA of GPER, then exposed to isoflavones or E2 for 60 min after serum-starvation for 6 h. Lower panel: Changes in the cortical F-actin score index. Bars represent 50 μm. **(C)** Western blot analysis of talin-1, vinculin, α-actinin, ERα, β-actin, cortactin, paxillin, and GAPDH levels after 60 min exposure of isoflavones or E2 in C6 cells. Representative blots are shown in the left panel, whereas quantitative analysis results are shown in the right panel. Data are expressed as mean ± SEM and are representative of at least three independent experiments. ****p < 0.0001, ***p < 0.001, **p < 0.01, *p < 0.05, indicates statistical significance measured using Bonferroni’s test compared with control (-). ^####^ *p* < 0.0001, ^###^ *p* < 0.001, ^##^ *p* < 0.01, ^#^ *p* < 0.05, indicates statistical significance measured using Bonferroni’s.

The ability of cells to migrate requires complex molecular events that are initiated by the assembly of F-actin to alter the cellular morphology to move through interstitial submicron size pores in tissues (40, 41). In order to further examine the mechanisms involved in isoflavone-induced acceleration of cell migration, we visualized F-actin with Phalloidin-iFlour 594 reagent. At first, we examined filopodia formation using FiloQuant by ImageJ Fiji. Isoflavones or E2 exposure for 30 min increased filopodia formation (**Figure 3A**, upper panel). Quantitative analysis showed the increase in the number and length of the filopodia (**Figure 3A**, lower panel). Then we continued to examine the formation of stress fibers in the cortical actin filaments using cortical F-actin score (CFS) index. The CFS index was determined based on F-actin cytoskeletal reorganization for each cell. It was scored on a scale ranging from 0 to 3, based on the degree of cortical F- actin ring formations 0, no cortical F-actin, normal stress fibers; 1, cortical F-actin deposits below half the cell border; 2, cortical F-actin deposits exceeding half the cell border; and 3, complete cortical ring formatting and/or total absence of central stress fiber. Isoflavones or E2 attenuated stress fibers to increase cortical actin filaments in astrocytes after 30-min exposure (**Figure 3B**, upper panel). The CFS index significantly increased after 10 nM exposure of isoflavone or E2 and these effects were reduced by knockdown of GPER (**Figure 3B**, lower panel). In addition, we also examined the effects of isoflavones and E2 on focal adhesion proteins related to actin reorganization. We found that 10 nM isoflavones or E2 increased the protein expression levels of vinculin, cortactin and paxillin, and these effects were reduced by knockdown of GPER (**Figure 3C**). We also found that both isoflavones and E2 increased the protein expression levels of ERα and it reduced after silencing the GPER. However, there is no significant changes in the talin-1 and α-actinin protein expression levels after the exposure of isoflavones or E2 (**Figure 3C**). These results indicate the exposure of isoflavones induced F-actin formation, which may have accelerated the migration of astrocytes.

### Isoflavones Accelerated Cell Migration *via* the GPER/PI3K/FAK/Akt Pathway

**Figure 4:**
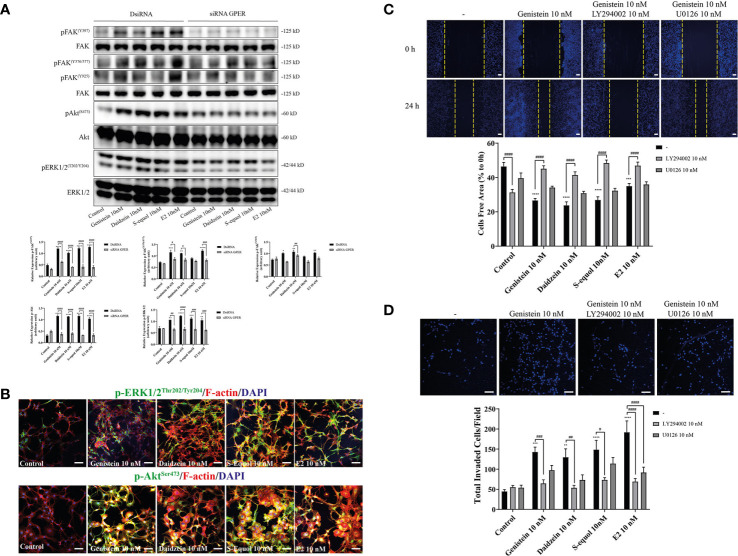
Effects of isoflavones on activation of the PI3K/FAK/Akt axis. **(A)** Western blot analysis of p-FAK, FAK, pERK1/2, ERK1/2, pAkt, and Akt levels after 30 min exposure of isoflavones or E2 in the astrocytes that have been transfected with DsiRNA or short interfering RNA (siRNA) G-protein-coupled estrogen receptor (GPER). Representative blots are shown in the upper panel, whereas quantitative analysis results are shown in the lower panel. **(B)** Representative photomicrographs showing immunocytochemistry results for pERK1/2 and pAkt, with F-actin and DAPI staining to examine the effects of isoflavones or E2 in C6 cells. C6 clonal cells were exposed to isoflavones for 30 min after serum starvation for 6 h. **(C)** Upper panel: Representative photomicrographs showing the effect of LY294002, a PI3K inhibitor, and/or U0126, an ERK1/2 inhibitor, on genistein-accelerated C6 cell migration measured using wound healing assay. Lower panel: Quantitative analysis of the effect of LY294002 or U0126 on isoflavones-accelerated C6 cell migration. **(D)** Upper panel: Representative photomicrographs showing the effect of LY294002, a PI3K inhibitor, and/or U0126, an ERK1/2 inhibitor, on genistein-accelerated C6 cell migration measured using cell invasion assay. Lower panel: Quantitative analysis of the effect of LY294002 or U0126 on isoflavones-accelerated C6 cell invasion. Bars represent 50 μm. Data are expressed as mean ± SEM and are representative of at least three independent experiments. ****p < 0.0001, ***p < 0.001, **p < 0.01, *p < 0.05, indicates statistical significance measured using Bonferroni’s test compared with control (-). ^####^ *p* < 0.0001, ^###^ *p* < 0.001, ^##^ *p* < 0.01, ^#^ *p* < 0.05, indicates statistical significance measured using Bonferroni’s.

Activation of GPER can induce FAK, Akt, and ERK phosphorylation signaling. To examine the downstream targets of GPER activation by isoflavones, we performed Western blot analysis to measure phosphorylation of FAK, Akt, and ERK1/2 after knockdown of GPER. Isoflavones or E2 increased pFAK, pAkt, and pERK1/2 protein levels, and these effects were reduced after knockdown of GPER (**Figure 4A**). Moreover, immunofluorescence study showed increased F-actin expression concurrent with pAkt, but not with pERK1/2 (**Figure 4B** and **Figure SI.2**). To examine further, we cultured cells with isoflavones and either PI3K inhibitor (LY294002) or ERK1/2 inhibitor (U0126) prior to performing wound healing and invasion assays. LY294002 suppressed isoflavones or E2-accelerated cell migration in C6 cells. No significant effects were observed after co-exposure of isoflavones with U0126 (**Figures 4C, D**). However, we found significant difference in cell invasion after co-exposure of E2 and U0126 (**Figure 4D**). These results indicate isoflavones increased cell migration *via* GPER/PI3K/FAK/Akt pathway.

### Isoflavone Activated p-Akt Led to Increase RhoGTPase Levels

**Figure 5:**
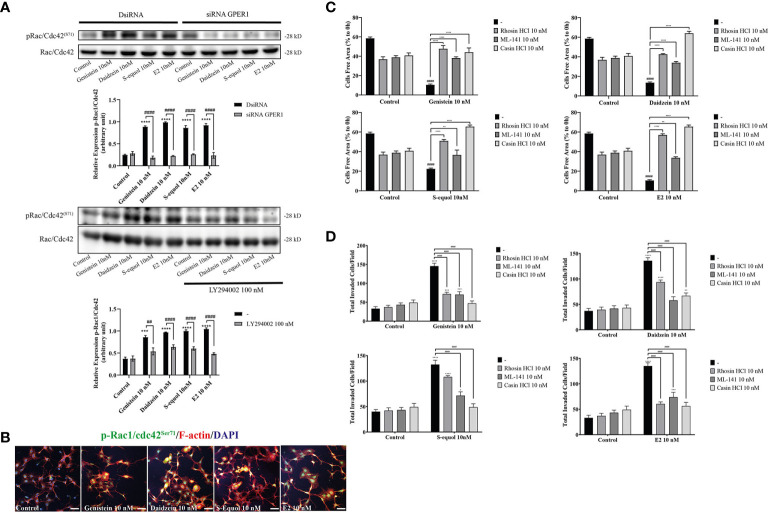
Effects of isoflavones on phosphorylation of RhoGTPase and their involvement on cell migration and F-actin formation. **(A)** Western blot analysis for pRac1/Cdc42 and Rac1/Cdc42 levels after 30 min exposure of isoflavones or E2 in the astrocytes that have been transfected with DsiRNA or short interfering RNA (siRNA) G-protein-coupled estrogen receptor (GPER), or co-exposure with LY194002. Representative blots are shown in the upper panel, whereas quantitative analysis results are shown in the lower panel. **(B)** Representative photomicrographs showing immunocytochemistry results for pRac1/Cdc42 with F-actin and DAPI staining to examine the effects of isoflavones or E2 in astrocytes. Astrocytes were exposed to isoflavones for 60 min after serum starvation for 6 h. **(C)** Quantitative analysis of the effect of rhosin HCl (RhoA inhibitor), ML-141 (Rac1/Cdc42 inhibitor), and casin HCl (Cdc42 inhibitor) on isoflavone-accelerated cell migration measured by wound healing assay. **(D)** Quantitative analysis of the effect of rhosin HCl (RhoA inhibitor), ML-141 (Rac1/Cdc42 inhibitor), and casin HCl (Cdc42 inhibitor) on isoflavone-accelerated cell migration measured by cell invasion assay. Bars represent 50 μm. Data are expressed as mean ± SEM and are representative of at least three independent experiments. ****p < 0.0001, ***p < 0.001, **p < 0.01, *p < 0.05, indicates statistical significance measured using Bonferroni’s test compared with control (-). ^####^p < 0.0001, ^###^ *p* < 0.001, ^##^ *p* < 0.01, indicates statistical significance measured using Bonferroni’s.

Cell movement is depended on the involvement of Rho GTPase activation on actin. RhoA, Rac1, and Cdc42 play major roles in actin polymerization that leads to cell movement. We examined the effects of isoflavones on Rho GTPase signaling using Western blot, wound healing assay, and immunocytochemistry analyses. Western blot analysis showed that 10 nM isoflavones or E2 increased protein levels of pRac1/Cdc42 (**Figures 5A, B** and **Figure SI. 3**). The phosphorylation of Rac1/Cdc42 significantly decreased after knockdown of GPER or co-exposure with LY294002 (**Figure 5A**). Immunocytochemistry analysis also showed an overlap between F-actin and pRac1/Cdc42 (**Figure 5B**). Co-exposure with RhoA inhibitor (rhosin), Rac1/Cdc42 inhibitor (ML-141), or Cdc42 inhibitor (chasin) significantly suppressed isoflavone or E2-accelerated cell migration in the wound healing and cell invasion assays (**Figures 5C, D**). These results indicate that exposure to isoflavones increased the expression levels of Rho GTPase to induce F-actin formation and subsequent activation of cell motility.

### Potential Binding of Isoflavones to the GPER

**Figure 6:**
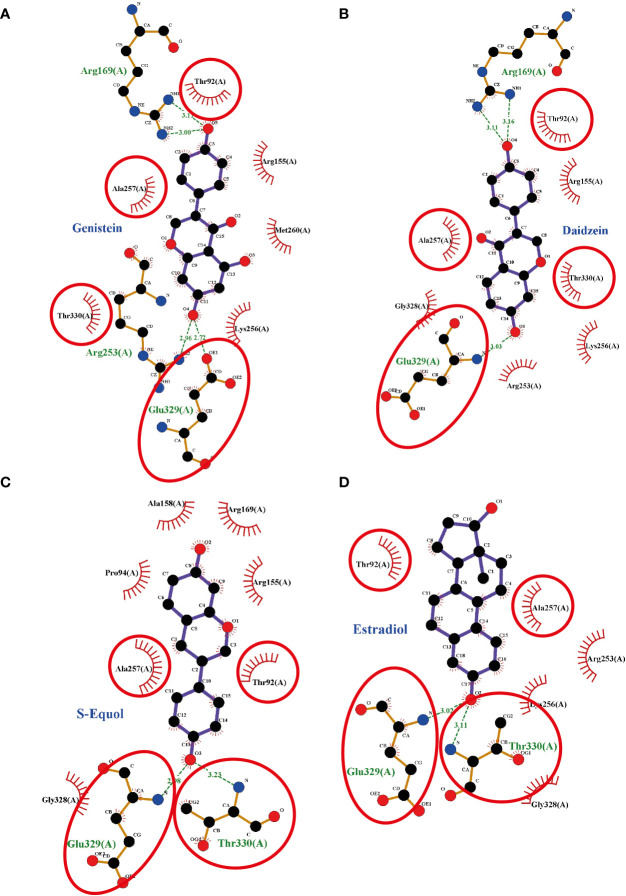
Interaction plots between **(A)** genistein, **(B)** daidzein, **(C)** S-equols, or **(D)** E2 and the G-protein-coupled estrogen receptor (GPER) generated by LigPlot^+^ v.2.0, with each subsequent plot being automatically fitted. Red circles and ellipses in each plot indicate amino acid residues that have equivalent 3D positions with respect to the residues in the first plot. Hydrogen bonds are shown as green dotted lines, while spooked arcs represent residues making nonbonded contacts with the ligand.

To investigate the plausible binding modes of isoflavones to GPER, we generated *in silico* binding models using molecular docking study with AutoDocks Vina. Since the crystal structure of GPER remains unknown, the 3D protein structure was predicted using the I-TASSER website. The encoding sequence for GPER was retrieved from the UniProt database (accession number Q6FHU6) and submitted to the I-TASSER website, a specialized server for building 3D models of seven transmembrane receptors. The I-TASSER server yielded five predicted models from 10 different templates. Before generating the MD simulations, several geometrical observables such as area per lipid, the root mean square deviation (RMSD) of heavy atom with respect to the starting conformation, and the atomic fluctuation were evaluated to observe if the systems reached equilibrium. RMSD is known standards for measuring structural similarity between two structures which are usually used to measure the accuracy of structure modeling. MD simulations shows that after reached the equilibrium, the RMSD values of 8.4 ± 4.5, 5.6 ± 0.19, 5.5 ± 1.2, 5.0 ± 0.13, and 5.9 ± 1.1 Å for GPER, GPER-genistein, GPER-daidzein, GPER-S-equol, and GPER-E2, respectively. The 3D docking results of GPER showed isoflavones and E2 possess a similar binding pose under blind docking procedures. Isoflavones and E2 could form a hydrogen bond with Glu 329 and have the same amino acid residues that have equivalent 3D positions concerning the residues in the first plot, as shown in red circles and ellipses (**Figures 6A–D**). An additional possibility to form hydrogen bond was also found between genistein and Arg 169 and Arg 253 (**Figure 6A**), daidzein and Arg 169 (**Figure 6B**), S-equol and Thr 330 (**Figure 6C**), and E2 and Thr 330 (**Figure 6D**). The binding affinities for genistein, daidzein, S-equol and E2 were -8.8, -8.6, -8.9, and -8.3 kcal/mol, respectively, with GPER. The docking poses in 3D model of isoflavones and E2 bound to GPER also shown in SI. 4. These results indicate that isoflavones may bind directly to GPER to accelerate cell migration.

## Discussion

The present study examined the effects of isoflavones and E2 on glial cell migration. Previously we reported that S-equol, a daidzein metabolite, activates GPER to induced F-actin rearrangement lead to increase astrocyte migration during cerebellar development with mechanisms remains unclear (14). This study reveals a novel mechanism of isoflavones (genistein, daidzein, and S-equol) in cell migration *via* GPER that may play an important role not only during brain development but also brain injury. We found that isoflavones increased 2D and 3D glial cell migration of primary astrocytes and C6 clonal cells. Isoflavone-accelerated cell migration was suppressed by knock down of GPER expression or coexposure with a GPER inhibitor. Isoflavone exposure also increased phosphorylation of FAK and Akt, which is a downstream target of GPER, leading to increased phosphorylation levels of RhoGTPase signaling, including Rac1 and Cdc42 that play a major role in F-actin formation. *In silico* analysis revealed that these isoflavones may directly interact with GPER. Our results showed the novel action of isoflavones in promoting glial cell migration *via* GPER signaling pathway.

The estrogenic activity of isoflavones has been well demonstrated (16, 42). Genistein exhibits >20-fold higher affinity for ERβ than ERα. Binding of isoflavones to ERs leads to shuttling of the ligand–ER complex to the nucleus and induces the transcription of target genes *via* the classical genomic pathway (16). In addition, recent studies have shown that isoflavones can interact with GPER and mediate rapid cellular signaling in neurons and endothelial cells (43–45). The present study also showed that the effects of isoflavones may be exerted by binding to the GPER to accelerate cell migration, since GPER knock down or co-exposure with GPER antagonist, at least in part, inhibited its action on astrocytes migration. On the other hand, knock down of nuclear ERs led to a weaker effect than that seen after GPER knockdown. These results indicate that the accelerated migration of astrocyte by isoflavones is mainly exerted *via* the GPER. The action may be slightly different to that of E2, since E2 action was inhibited by knock down of both nuclear ERs and GPER. We were unable to clarify the mechanisms involved in these differences. One possibility may be due to the difference in the affinities of the ER and GPER. The finding of our *in silico* study revealed a higher affinity of isoflavones compared with E2. However, further studies that include crystallization of GPER are required to confirm this difference.

The GPER belongs to the family of seven transmembrane-spanning GPCR and specifically binds estrogens, thereby activating intracellular signaling cascades (15, 16). In addition to cell migration, the GPER regulates various cellular functions, such as apoptosis, autophagy, proliferation, and differentiation, *via* a wide variety of signal transduction pathway including Ras/ERK (46, 47), PI3K/Akt (47–49), receptor tyrosine kinase (16), PLC-mediated pathway (50), and cAMP-mediated pathway (16). GPER induces rapid cellular effects including the production of cAMP, the mobilization of intracellular calcium, and the activation of kinase, such as ERK and PI3K, as well as ion channels and endothelial nitric oxide synthase (eNOS) (16). In addition, ERs (especially ERα) also activate such pathways. However, in cells that express both ERα and GPER there is a possibility of crosstalk or squelching between receptors (16, 51). The GPER also mediates estrogenic regulation of actin polymerization involves SRC-1 and PI3K/mTORC2 pathways in the hippocampus of female mice (49). In addition, the GPER acts *via* the PLCβ-PKC and Rho/ROCK/LIMK/cofilin pathways to regulate F-actin cytoskeleton assembly, thereby enhancing TAZ nuclear localization and activation, leading to increased cell migration and invasion (50). These varied GPER-activated pathways to regulate diverse cellular functions indicate the profound implications of GPER under physiological and pathophysiological conditions. In the present study, although isoflavones increased phosphorylation of ERK1/2 and Akt, inhibition of the PI3K/Akt pathway significantly suppressed the cell migration of astrocytes. These results indicate that, although various signal transduction pathways may be activated by isoflavones *via* the GPER, the Akt signaling pathway plays a major role in accelerating cell migration. Each signal transduction pathway of GPER may play a distinct role in cellular function.

The small GTPases of the Rho family (RhoA, Rac1 and Cdc42) appear to be at the heart of the initial signals leading to cellular polarization, and stress fiber, filopodia, and lamellipodia formation in migrating cells (40, 41, 52). Classic RhoGTPases are regulated by the opposing actions of Rho-specific guanine nucleotide exchange factors (GEFs) and GTPase-activating proteins (53). PI3K activates Rac and Cdc42 *via* activation of PIP3-regulated GEFs, and inhibition of Cdc42 in Ras-transformed cells decreased Akt signaling, leading to reduced migration/invasion (54). In addition to PI3K activates Rac and Cdc42, FAK also can influence the activity of RhoGTPases through a direct interaction or phosphorylation of the protein activators or inhibitors of RhoGTPases (55). It has been reported that estrogen activated FAK tyrosine phosphorylation (Tyr^397/576/577^) *via* Src, then regulated Cdc42 and Cdc42 effector Wiskott-Aldrich syndrome protein N-WASP (Neuronal-WASP) (56). N-WASP is a scaffold protein that links upstream signals to activate of the Arp2/3 complex, leading to actin nucleation for the rapid formation of actin network at the leading edge of the cell (55, 56). It has been known that paxillin and cortactin are direct target of FAK in the regulation of focal adhesion dynamics to promotes cell motility or invasion (55). In the present study, it is highly possible that activation of the PI3K/Akt axis and FAK induced the activation of Rac1, Cdc42, and focal adhesion protein, leading to accelerated cell migration. Cdc42 is essential for the formation of protrusions leading to elongated morphology. Deletion of Cdc42 in astrocytes revealed that the cells were still able to form protrusions, but in a nonoriented manner (26). Consequently, astrocytes failed to migrate in a directed manner toward a scratch. On the other hand, Rac is essential for both the development and maintenance of protrusions during migration. Rac1 also plays a role in local restructuring of the cytoskeleton coordinate with surface expansion, leading to astrocyte stellation (57). Isoflavones activated of Rac1/Cdc42 and increased the filopodia and cortical actin (**Figure 5**). Moreover, coexposure of isoflavones with ML-141, a Rac1/Cdc42 inhibitor, or casin, a Cdc42 inhibitor, significantly suppressed isoflavones-accelerated cell migration, indicating the involvement of Rac1/Cdc42 in this process. In addition, rhosin, a RhoA inhibitor also suppressed migration, indicating its involvement. These results are consistent with our hypothesis that isoflavones bind to the GPER (**Figure 6**) and activate PI3K/Akt axis signaling pathways to induce activation of RhoGTPase, resulting in F-actin formation and activation of astrocytes cell migration.

Astrocytes contribute to physiological brain function on many levels, including monitoring normal function of neurotransmitter uptake, synapse formation, regulation of the blood–brain barrier, and development of the CNS (21, 25). Astrocytes become dynamic migratory cells under certain physiological action and/or pathological conditions (21, 26, 40). Astrocyte migration requires coordination of complex signaling pathways, such as actin polymerization, delivery of membrane to the leading edge, and formation of attachments at the leading edge to provide traction, contraction, and disassembly of attachment at the rear (21, 26). Cell migration also depends on the mechanical and chemical interaction between the cells and their extracellular environment. Mechanical interaction depends on the polarization, adhesion, deformability, contractility, and proteolytic ability of cells (58). Our study showed that exposure to isoflavones increased the 2D or 3D migration of astrocytes by activating F-actin formation. Filopodia and stress fiber formation significantly increased after the exposure to isoflavones. F-actin in migrating cells are polarized with their plus (barbed)-ends toward the cell periphery against the plasma membrane, resulting in the formation of filopodia or lamellipodia, anchored focal adhesion, and extension at the front of the cells (40, 59). Filopodia are thin, finger-like, highly dynamic actin–rich membrane protrusions that extend out from the cell edge, thus extension of filopodia is driven by linear polymerization of actin filaments (41). During 3D migration, the mode of migration depends on the extracellular environment in which cells adopt round, amoeboid shapes, and extend lamellipodia. Extensive studies have revealed that amoeboid migration does not require focal adhesion-dependent force transmission, but instead relies on the global retrograde flow of cortical actomyosin (40). Cortical actin networks are involved in aligning along cell–cell junctions, supporting both stable and dynamics contacts in stationary epithelial and during collective cell migration (58). In summary, isoflavone-induced cell migration and F-actin rearrangement are processes that cannot be separated. However, further studies are required to understand how isoflavones induce cell migration as a result of the F-actin rearrangement.

The effect of isoflavones on human health remains controversial. While studies into the use of phytoestrogens as dietary supplements have reported various health benefits, such as antioxidant, anti-inflammatory, anti-cancer, and neuroprotective effects, there is a concern about potential adverse effects as results of modulating or disrupting endocrine function (8, 16, 60). However, most studies showing adverse effects used a higher dose of soy isoflavones than those found in the plasma of the population who regularly consume soy. Isoflavone dose is crucial to examine the effects of isoflavones in human health. For example, isoflavone dose affects risk of cancer. It was demonstrated that genistein enhanced cell proliferation in MCF-7 cells at concentrations of 10–100 nM. However, at higher concentrations (> 20 µM), genistein inhibited MCF-7 cell growth (8, 61). The total isoflavones plasma concentration in the Asian population consuming a traditional diet, including soy-based food, is in the range of 525–775 nM. In contrast, the total isoflavones plasma concentration in European countries was found to be <10 nM in individuals with a non-vegetarian diet and 79–148 nM in those with vegetarian and vegan diet (62). For the practical application of isoflavones in human health, studies using doses of isoflavones that represent plasma concentrations should be undertaken. The dose of isoflavones used in the present study ranged from 1–100 nM. Since this was an *in vitro* study, the results cannot be compared with those from *in vivo* conditions. However, our highlight the novel possibility that isoflavones activate astrocyte, indicating that they can be a useful supplementary compound during brain development or in the injured brain.

**Figure 7:**
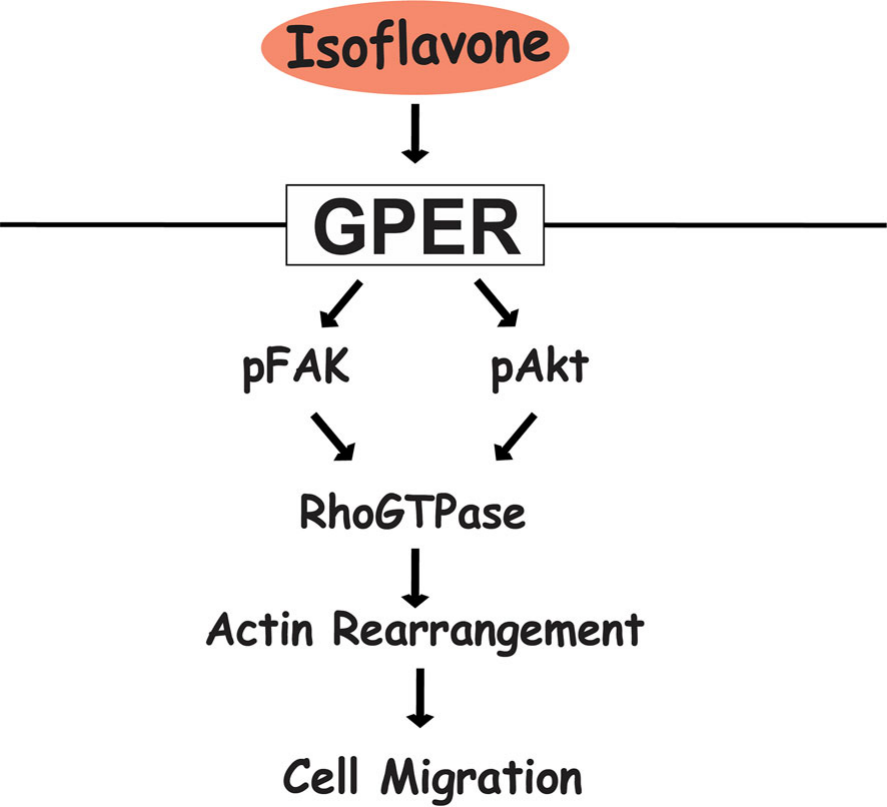
Proposed mechanism of isoflavone action on astrocyte migration *via* G-protein-coupled estrogen receptor (GPER). Isoflavones bind to the GPER and activate the PI3K/Akt and FAK signaling pathway, leading to phosphorylation of Rac1/Cdc42 and resulting in actin polymerization and formation of filopodia and stress fibers to promote cell migration in astrocytes.

In summary, our results showed that exposure of physiological concentrations of isoflavones increased cell migration *via* direct binding to GPER and subsequent activation of the PI3K/FAK/Akt/RhoGTPase signaling pathway, which induces F-actin formation (**Figure 7**). The present study highlights the potential use of isoflavones as an effective supplement to promote astrocyte migration during brain development or brain injury.

## Data Availability Statement

The raw data supporting the conclusions of this article will be made available by the authors, without undue reservation.

## Ethics Statement

The animal study was reviewed and approved by Animal Care and Experimentation Committee, Gunma University (19-024, 17 December 2018).

## Author Contributions

WA designed and performed the experiments, analyzed the results, and wrote the manuscript. IA and KH performed some of the experiments and analyzed the results. WM, TS, and NK designed the experiments, evaluated the data, and revised the manuscript. All authors contributed to the article and approved the submitted version.

## Funding

This work was supported in part by Grants-in-Aid for Scientific Research (nos. 18H03379 to NK, 16K00557 to WM, and 18J23449 to WA) from the Japanese Ministry of Education, Culture, Sports, Science and Technology (MEXT).

## Conflict of Interest

NK received research funding support from Otsuka Pharmaceutical Co., Ltd., Tokyo, Japan.

The remaining authors declare that the research was conducted in the absence of any commercial or financial relationships that could be construed as a potential conflict of interest.
