# Supplementary material for: Soy Isoflavones Accelerate Glial Cell Migration via GPER-Mediated Signal Transduction Pathway
Source: Front Endocrinol (Lausanne). 2020 Nov 4;11:554941. doi: 10.3389/fendo.2020.554941 (PMC7672195; doi:10.3389/fendo.2020.554941)
Supplement: Supplementary file 1 [file DataSheet_1.pdf]

Supplementary Figure Legends

SI 1. Isoflavones effects in astrocytes after siRNA transfection. Mouse primary cerebral cortex astrocytes were cultured for seven days prior to siRNA transfection, and matrigel invasion assay followed by DAPI staining was performed. Upper panel: Representative photomicrographs showing the effects of isoflavone on 3D matrigel invasion assays using astrocytes after the transfection of scRNA, siRNA GPER, ERα, or ERβ. Cell nuclei were stained with DAPI. Bars represent 50 μm. Middle panel: The change in GPER, ERα, or ERβ mRNA expression levels in astrocytes after siRNA transfection. All experiments were repeated 3 times, using independent RNA preparations to confirm the consistency of the results. The mRNA levels were normalized by the mRNA level of GAPDH. Lower panel: List of qRT-PCR primer sequences.

SI 2. Effects of isoflavones on phosphorylation of ERK1/2 and Akt. C6 clonal cells were exposed to isoflavones for 30 min after serum starvation for 6 h. Upper panel: Representative photomicrographs showing immunocytochemistry results of C6 cells. After fixation, cells were incubated with rabbit anti-phospho-p44/42 MAPK (ERK1/2) (Thr202/Tyr204) (1:200; Cell Signaling) antibody, followed by incubation with donkey anti-rabbit IgG (H+L) secondary antibody, Alexa Fluor 488 conjugate (1:200; Thermo Fisher Scientific) and CytoPainter Phalloidin-iFluor 594 reagent (AbCam). The cell nuclei were also stained with DAPI. Cells were then inspected under a laser confocal scanning microscope (ZEISS LSM 880, Carl Zeiss Microscopy GmbH, Jena, Germany). Bars represent 50 μm. Lower panel: Representative photomicrographs showing immunocytochemistry results of C6 cells. After fixation, cells were

incubated with rabbit monoclonal anti-phospho-Akt (Ser473) (D9E) XP (1:200; Cell Signaling) antibody, followed by incubation with donkey anti-rabbit IgG (H+L) secondary antibody, Alexa Fluor 488 conjugate (1:200; Thermo Fisher Scientific) and CytoPainter Phalloidin-iFluor 594 reagent (AbCam). The cell nuclei were also stained with DAPI. Cells were then inspected under a laser confocal scanning microscope (ZEISS LSM 880, Carl Zeiss Microscopy GmbH, Jena, Germany). Bars represent 50 μm.

SI 3. Effects of isoflavones on phosphorylation of Rac1/Cdc42. C6 clonal cells were exposed to isoflavones for 30 min after serum starvation for 6 h. Upper panel: Representative photomicrographs showing immunocytochemistry results C6 cells. After fixation, cells were incubated with rabbit anti-phospho-Rac1/Cdc42 (Ser71) (1:200; Cell Signaling) antibody, followed by incubation with donkey anti-rabbit IgG (H+L) secondary antibody, Alexa Fluor 488 conjugate (1:200; Thermo Fisher Scientific) and CytoPainter Phalloidin-iFluor 594 reagent (AbCam). The cell nuclei were also stained with DAPI. Cells were then inspected under a laser confocal scanning microscope (ZEISS LSM 880, Carl Zeiss Microscopy GmbH, Jena, Germany). Bars represent 50 μm.

SI 4. Isoflavones and E2 can bind to GPER binding pocket. Results of the docking pose in 3D models of genistein (B), daidzein (C), S-equol (D), and E2 (E) in the same binding pocket in GPER. A blind docking strategy was utilized to include all possible ligand binding sites.
