# Supplementary figures and images for: Soy Isoflavones Accelerate Glial Cell Migration via GPER-Mediated Signal Transduction Pathway
Source: Front Endocrinol (Lausanne). 2020 Nov 4;11:554941. doi: 10.3389/fendo.2020.554941 (PMC7672195; doi:10.3389/fendo.2020.554941)

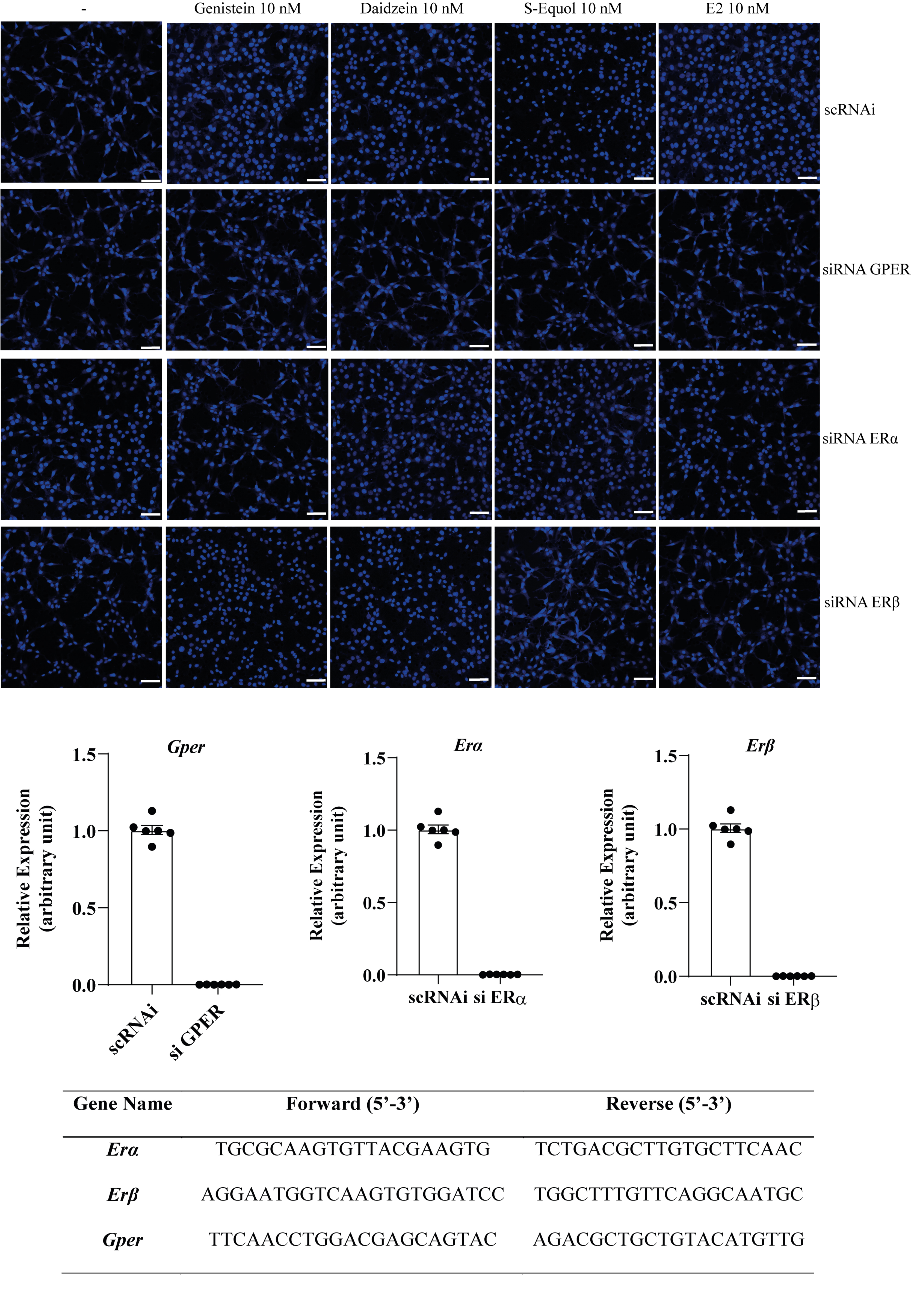

Supplement: Supplementary file 2 [file Image_1.jpeg]

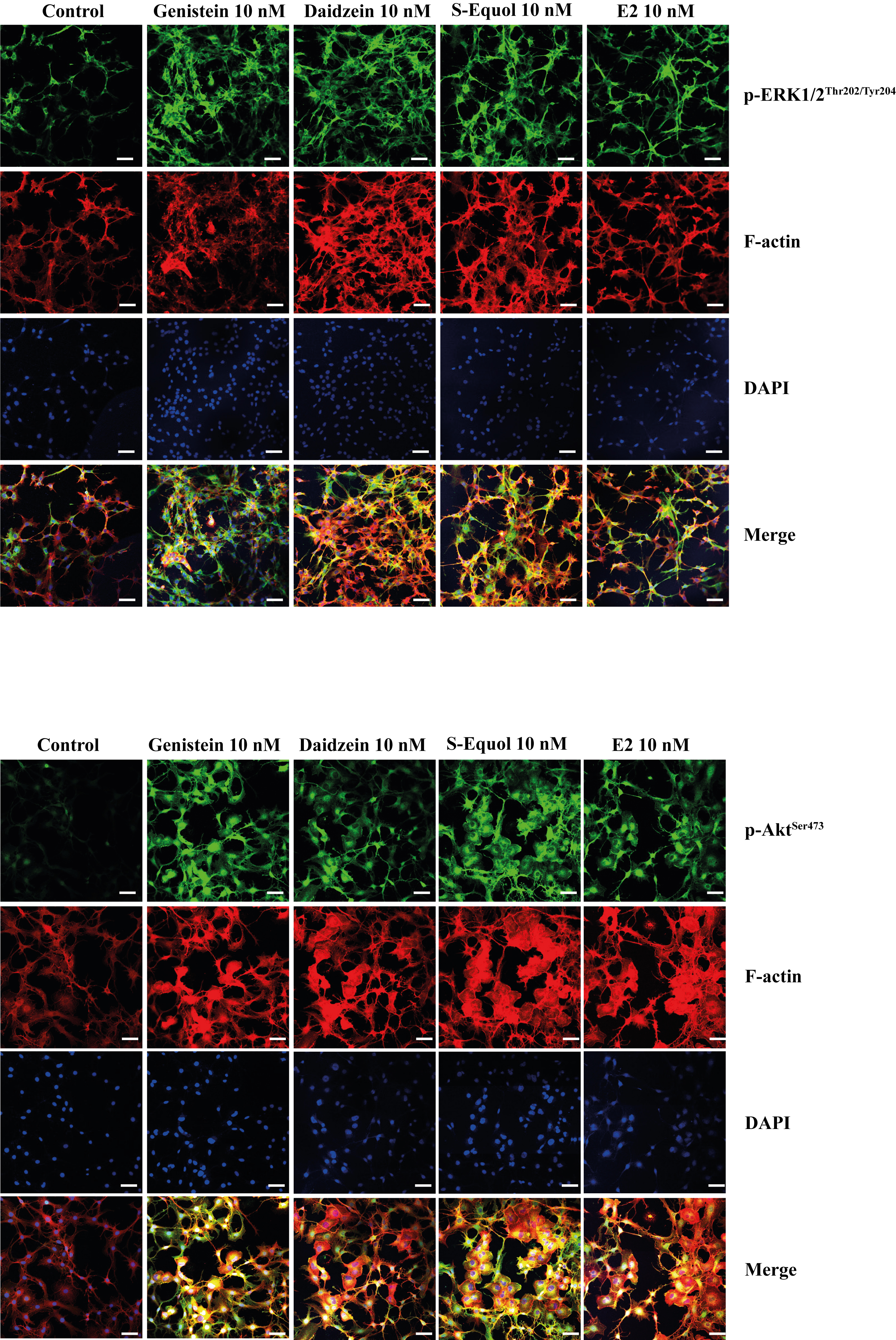

Supplement: Supplementary file 3 [file Image_2.jpeg]

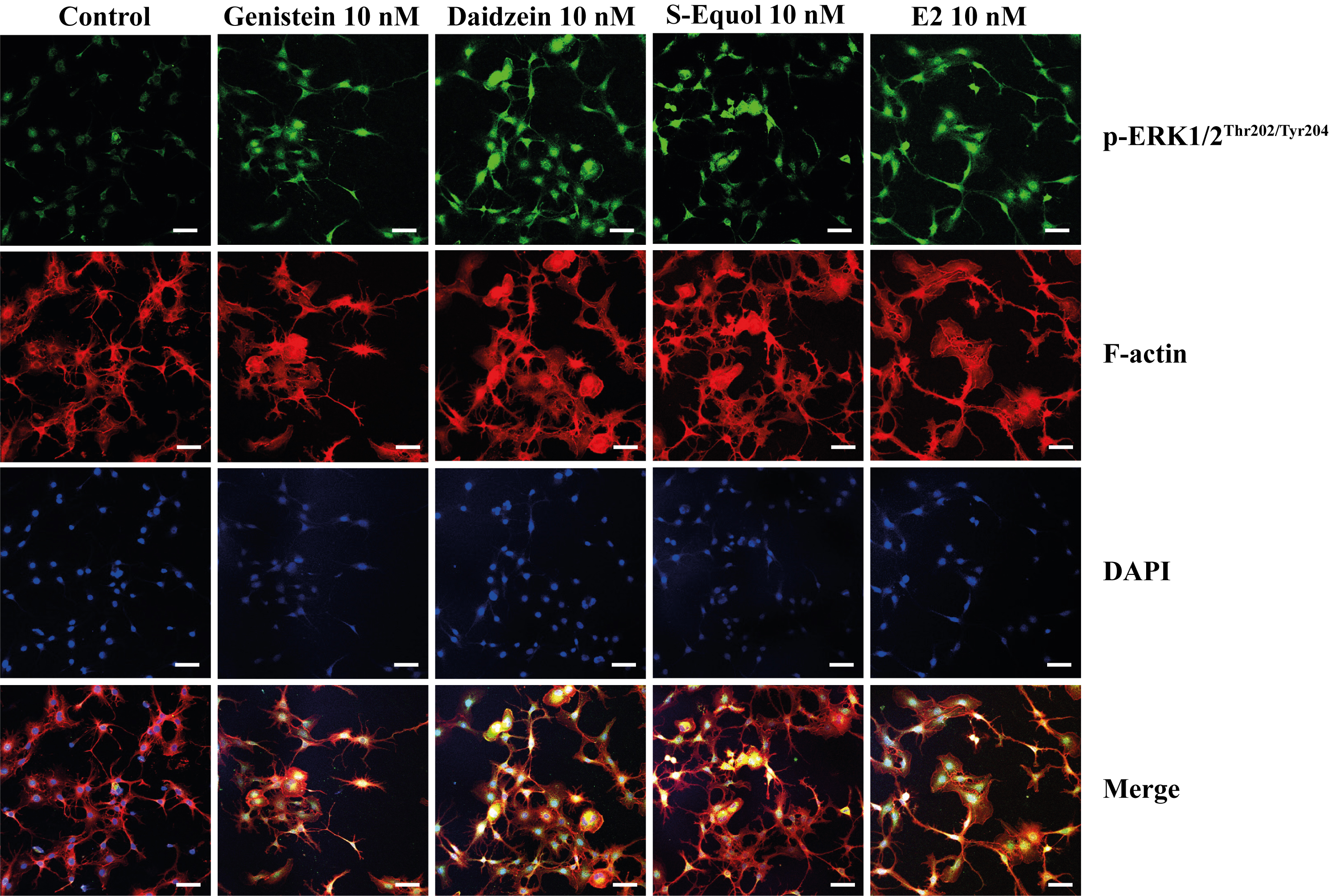

Supplement: Supplementary file 4 [file Image_3.jpeg]

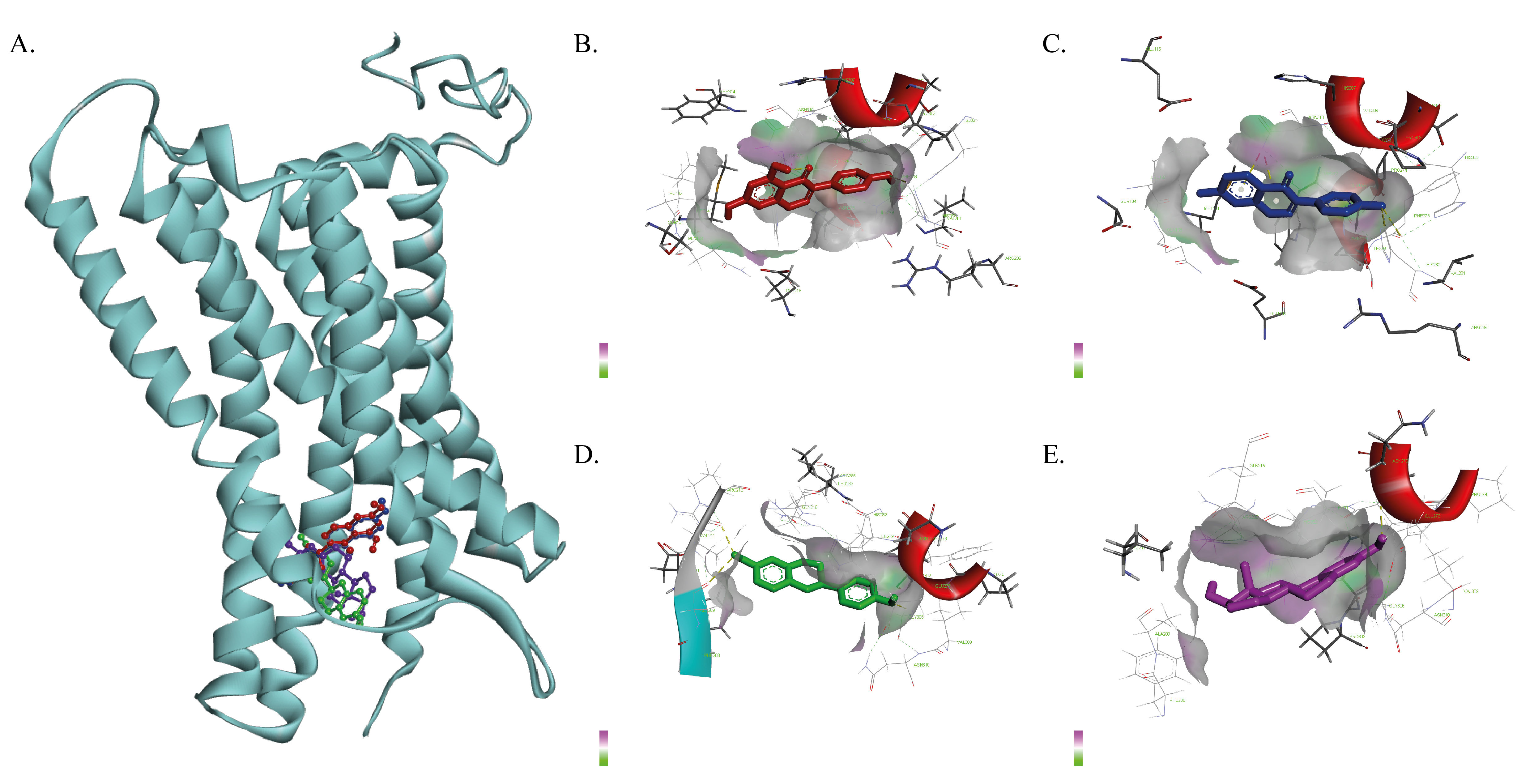

Supplement: Supplementary file 5 [file Image_4.jpeg]
